# Evolution of *Mycobacterium tuberculosis* drug resistance in the genomic era

**DOI:** 10.3389/fcimb.2022.954074

**Published:** 2022-10-07

**Authors:** Camus Nimmo, James Millard, Valwynne Faulkner, Johana Monteserin, Hannah Pugh, Eachan Oliver Johnson

**Affiliations:** ^1^ Systems Chemical Biology of Infection and Resistance Laboratory, Francis Crick Institute, London, United Kingdom; ^2^ Institute of Infection and Global Health, University of Liverpool, Liverpool, United Kingdom

**Keywords:** TB, acquired resistance, within-host evolution, clonal expansion, compensatory mutations, bedaquiline, delamanid, pretomanid

## Abstract

*Mycobacterium tuberculosis* has acquired drug resistance to all drugs that have been used against it, including those only recently introduced into clinical practice. Compared to other bacteria, it has a well conserved genome due to its role as an obligate human pathogen that has adapted to a niche over five to ten thousand years. These features facilitate reconstruction and dating of *M. tuberculosis* phylogenies, giving key insights into how resistance has been acquired and spread globally. Resistance to each new drug has occurred within five to ten years of clinical use and has occurred even more rapidly with recently introduced drugs. In most cases, resistance-conferring mutations come with a fitness cost, but this can be overcome by compensatory mutations which restore fitness to that of wild-type bacteria. It is likely that *M. tuberculosis* acquires drug resistance while maintaining limited genomic variability due the generation of low frequency within-host variation, combined with ongoing purifying selection causing loss of variants without a clear fitness advantage. However, variants that do confer an advantage, such as drug resistance, can increase in prevalence amongst all bacteria within a host and become the dominant clone. These resistant strains can then be transmitted leading to primary drug resistant infection in a new host. As many countries move towards genomic methods for diagnosis of *M. tuberculosis* infection and drug resistance, it is important to be aware of the implications for the evolution of resistance. Currently, understanding of resistance-conferring mutations is incomplete, and some targeted genetic diagnostics create their own selective pressures. We discuss an example where a rifampicin resistance-conferring mutation which was not routinely covered by standard testing became dominant. Finally, resistance to new drugs such as bedaquiline and delamanid is caused by individually rare mutations occurring across a large mutational genomic target that have been detected over a short time, and do not provide statistical power for genotype-phenotype correlation – in contrast to longer-established drugs that form the backbone of drug-sensitive antituberculosis therapy. Therefore, we need a different approach to identify resistance-conferring mutations of new drugs before their resistance becomes widespread, abrogating their usefulness.

## Introduction

*Mycobacterium tuberculosis* is an ancient bacterial pathogen that has acquired drug resistance to all drugs that have been used against it, despite lacking several key mechanisms available to other bacteria to facilitate rapid spread of resistance such as horizontal gene transfer and mobile resistance elements. In the absence of such mechanisms, all antituberculosis drug resistance is conferred by genomic mutations, mostly single nucleotide polymorphisms (SNPs), that are propagated through replication of resistant bacteria and onward transmission. In this review, we discuss how *M. tuberculosis* can develop drug resistance despite maintaining a comparatively well-conserved genome compared to other bacterial pathogens (Eldholm and Balloux, 2016) and give examples of how it has acquired resistance at the between-host and within-host levels. Finally, we assess how diagnostics may be affected by resistance and shape its emergence, and we outline the implications for identifying resistance to new drugs against tuberculosis that are entering clinical use.

## Key features of the *Mycobacterium tuberculosis* genome

The whole genome sequence of H37Rv, originally isolated from a patient treated in New York in 1905 and now the most used laboratory strain of *M. tuberculosis*, was published in 1998 (Cole et al., 1998). The most recent annotation reports it as 4.4 megabases in length, making it 33% smaller than *Mycobacterium smegmatis* [also named *Mycolicibacterium smegmatis*, (Gupta et al., 2018), although the usefulness of this is contested (Tortoli et al., 2019)], and one of the smallest apart from *Mycobacterium leprae* (1.6 megabases). It contains 3906 coding genes, of which a large number are responsible for fatty acid metabolism due to the complex mycobacterial cell wall. It is very rich in guanine and cytosine residues, and unlike many other bacteria (e.g. gram-negatives) there is no evidence of recombination and no accessory genome (Eldholm and Balloux, 2016). About 10% of the genome is devoted to a characteristic set of proline (P)- and glutamate (E)-rich proteins called the PE and PPE gene families, which are heterogenous and consist of numerous tandem repeats and are hypothesised to be surface antigens that are responsible for interaction with the host immune system (Fishbein et al., 2015). They are difficult to resolve by short read sequencing and as a result have been historically excluded from many genomic analyses of *M. tuberculosis*.

*M. tuberculosis* has traditionally been viewed as a genetically homogenous bacterium that has evolved into a specialised human pathogen with a lower mutation rate than most other bacteria at 0.3 to 0.5 SNPs per genome per year (Eldholm and Balloux, 2016). The *M. tuberculosis* complex (MTBC) is likely to have originated from the transition of an environmental mycobacterial ancestor shared with the pathogen *M. canetti* (Soolingen et al., 1997). The transition came with a corresponding reduction in genome size and loss of the ability for genetic recombination or gene transfer, perhaps because it developed into a specialised pathogen that lives only in one ecological niche. The original divergence of the MTBC from environmental mycobacteria is likely to have happened in Africa and then been spread globally by human migration (Gagneux, 2018). Animal-adapted strains of the MTBC, including *M. bovis* (cows) and *M. caprae* (goats) are likely to have been transferred from humans as evidenced by comparative genomic studies that show loss of genes from *M. tuberculosis sensu stricto* to other members of the MTBC (Comas et al., 2013). Genetic evidence suggests that MTBC is likely to have originated around 5,000-10,000 years ago (Bos et al., 2014; Kay et al., 2015; Chiner-Oms et al., 2019), corresponding with archaeological evidence of *M. tuberculosis* DNA and lipids in skeletal remains from 9,000 years ago (Hershkovitz et al., 2008).

The modern MTBC comprises seven human-adapted lineages, which are phylogenetically distinct groups clades that have evolved separately, having diverged over a period of 500 to 3000 years (O’Neill et al., 2019) and several animal-adapted strains. Lineages 1, 2, 3, 4 and 7 are traditionally referred to as *M. tuberculosis sensu stricto*, while lineages 5 and 6 are known as *M. africanum*. Lineages 5 and 6 are restricted to West Africa and lineage 7 to East Africa, suggesting that they may have specifically adapted to their host populations (Asante-Poku et al., 2015). Lineage 1 to 4 are globally distributed, with lineages 2 and 4 being the most prevalent worldwide (Gagneux, 2018)

## Mechanisms of drug resistance

The majority of *M. tuberculosis* antibiotic resistance is conferred by genomic mutations – usually SNPs or small insertions or deletions, and occasionally larger deletions or inversions. Given the lack of horizontal gene transfer or episomal resistance genes (Boritsch et al., 2016), these generally arise spontaneously and are chromosomally encoded, with spread through replication within host and onward transmission between hosts of resistant bacteria.

In contrast to organisms which exhibit horizontal gene transfer and therefore can also acquire extrachromosomal drug-inactivating resistance genes, there are three main mechanisms through which antituberculosis drug resistance can be acquired: target-based mutations, activator mutations and modulation of efflux pumps. Target-based mutations are where the drug target itself becomes mutated, usually preventing drug binding. Many antituberculosis drugs are administered as prodrugs that require activation by bacterial enzymes to produce their active form. In these cases, mutations of drug activators can lead to resistance. Finally, efflux pumps may pump active drug out of the bacterial cell, although there are fewer examples of these. Examples of each of these mechanisms are shown in **Table 1** (Silva and Palomino, 2011; Dookie et al., 2018). Some drugs have multiple mechanisms of resistance, for example isoniazid resistance can be conferred by target-based (*inhA*) or activator (*katG*) mutations. Some mutations may lead to cross-resistance, while others monoresistance. For example, *atpE* is the target for only bedaquiline. However, resistance to bedaquiline, clofazimine, and even new tuberculosis inhibitors like BRD-9327 can be conferred by efflux pump regulator mutations in *Rv0678* (Johnson et al., 2020).

**Table 1:** Categories of mutation leading to *M. tuberculosis* drug resistance.

Category	Gene (drug)	Mechanism of resistance
Target-based	*rpoB* (rifampicin)	Rifampicin is unable to bind RNA polymerase, responsible for mRNA elongation (Telenti et al., 1993)
	*inhA* (isoniazid, ethionamide)	Both drugs unable to bind NADH-dependent enoyl-acyl carrier protein reductase responsible for mycolic acid synthesis (Banerjee et al., 1994)
	*gyrA/B* (fluoroquinolones)	Mutations prevent binding to DNA gyrase required for DNA replication (Takiff et al., 1994)
	*rrl* (aminoglycosides)	Prevent binding to 23S ribosomal RNA which prevents protein synthesis (Suzuki et al., 1998)
	*atpE* (bedaquiline)	Prevents binding to F1F0 proton ATP synthase, part of electron transport chain (Huitric et al., 2010)
	*embB* (ethambutol)	Mutations in the mycobacterial arabinosyl transferase enzyme preventing synthesis of arabinogalactan for the cell wall (Telenti et al., 1997)
Drug activator	*katG* (isoniazid)	Isoniazid is activated by the katG-encoded catalase-peroxidase enzyme. S315T mutations prevent activation while maintaining native gene function (Pym et al., 2002)
	*ethA* (ethionamide)	Mutations in the activating mono-oxygenase enzyme encoded by ethA, or its regulator ethR (Morlock et al., 2003)
	*pncA* (pyrazinamide)	Diverse range of mutations in pyrazinamide activating PZase encoded by pncA lead to resistance, including any loss of function mutation as PZase loss does not impair fitness (Scorpio and Zhang, 1996)
	*fbiA/B/C*, fgd1, ddn (delamanid/pretomanid)	Wide variety of mutations inactivating enzymes ddn and co-enzyme fgd1. Also mutations in synthetic pathway for F420 cofactor required for activation (fbiA/B/C) (Haver et al., 2015)
Efflux pumps	*Rv0678* (bedaquiline, clofazimine)	Mutations affecting or preventing function of the Rv0678 repressor of the MmpL5 efflux pump lead to overexpression of the pump and presumed efflux of bedaquiline and clofazimine (Hartkoorn et al., 2014)

## Factors affecting acquisition of drug resistance

**Figure 1:**
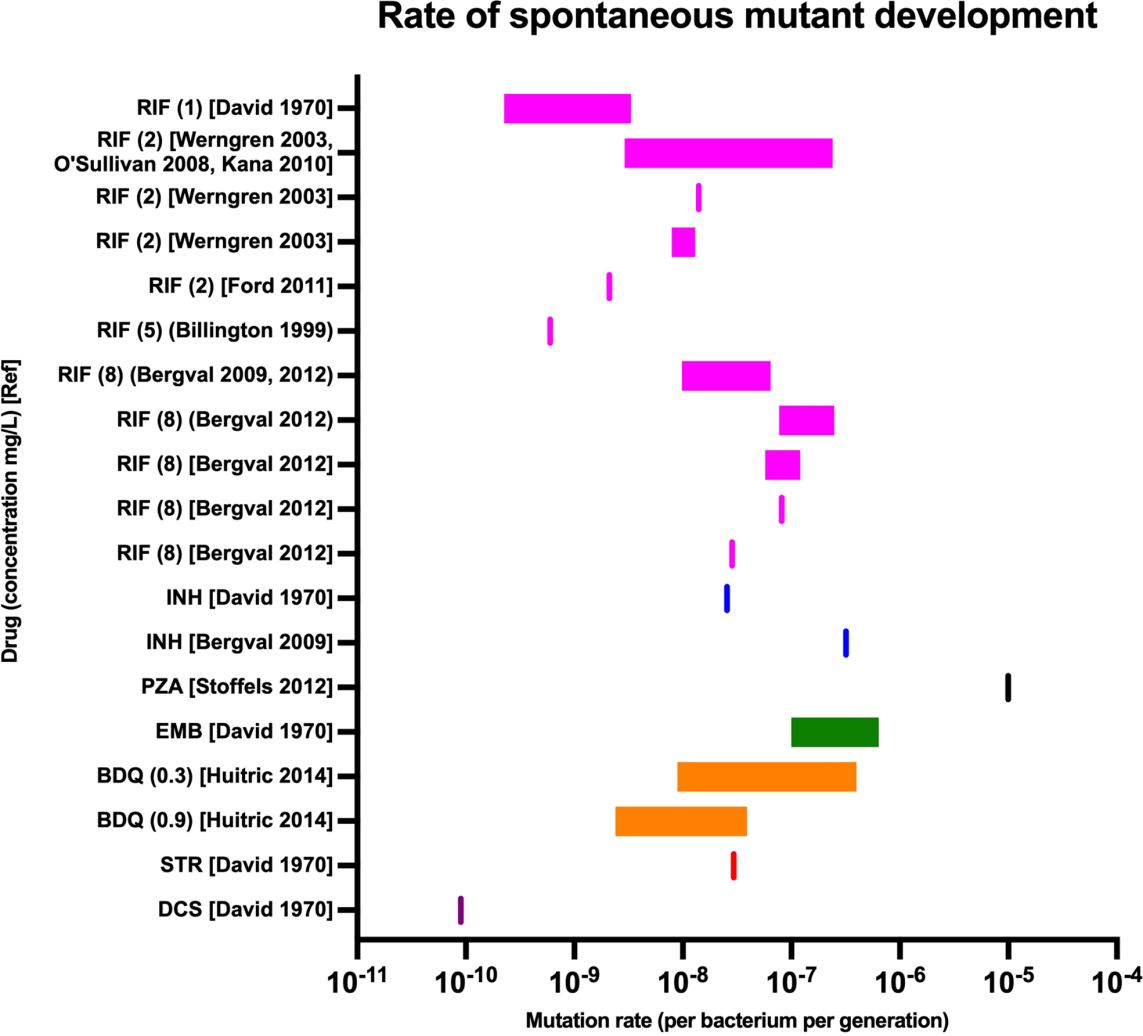
Spontaneous *in vitro* mutation rates for key antituberculous drugs (McGrath et al., 2014). Rates were measured in a variety of laboratory and clinical strains from varying bacterial lineages. Drug names abbreviated: RIF, rifampicin; INH, isoniazid; PZA, pyrazinamide; EMB, ethambutol; BDQ, bedaquiline; STR, streptomycin; DCS, D-cycloserine. Concentrations in mg/L shown in brackets followed by original reference (David, 1970; Billington et al., 1999; Werngren and Hoffner, 2003; O’Sullivan et al., 2008; Bergval et al., 2009, 2012; Huitric et al., 2010; Kana et al., 2010; Ford et al., 2011; Stoffels et al., 2012).

Prevalence of drug resistance varies by drug, patterns of drug usage (including the combinations of drugs it was used with), bacterial genetic background and country (Brynildsrud et al., 2018; Ektefaie et al., 2021). The ability of *M. tuberculosis* to acquire drug resistance to each drug is underpinned by the rate at which spontaneous mutants arise and survive. This is different for each drug, with pyrazinamide having a particularly high rate of resistance acquisition *in vitro* and rifampicin a lower rate (**Figure 1**) (McGrath et al., 2014; David, 1970; Billington et al., 1999; Werngren abd Hoffner, 2003; O'Sullivan et al., 2008; Bergval et al., 2009, 2012; Huitric et al., 2010; Kana et al., 2010; Ford et al., 2011; Stoffels et al., 2012). However, the clinical relevance of *in vitro* mutation rates is only one aspect of the likely robustness of a drug against the development of resistance against it. For example, the studies examining spontaneous development of bedaquiline-resistant mutants (Huitric et al., 2010) recorded the rate at which bacteria developed *atpE* mutations, which is the main gene determining bedaquiline resistance *in vitro* but not *in vivo*, where virtually all clinically reports mutations are in the *Rv0678* gene (Huitric et al., 2010). This is likely because *atpE* is an essential gene and mutations carry a high fitness cost *in vitro*, underlining the importance of understanding *in vivo* fitness costs of mutations.

Bacterial genetic background is also likely to affect the ability of certain strains to acquire drug resistance. The best described example of this is higher prevalence of drug resistance amongst lineage 2 strains compared to other bacterial lineages (Parwati et al., 2010; Ektefaie et al., 2021). It is likely that lineage 2 *M. tuberculosis* strains have a greater inherent ability to acquire drug resistance, and this has now been suggested by multiple studies (Ford et al., 2013; Hakamata et al., 2020; Nimmo et al., 2020; Ortiz et al., 2021), although at least one study did not find this link (Guerra-Assunção et al., 2015). While the mechanisms through which this may occur have not been fully elucidated, *in vitro* work with *M. smegmatis* (a related mycobacterium often used for laboratory studies) has shown that ribosomal mutations can lead to resistance to multiple antibiotics and enhanced bacterial survival (Gomez et al., 2017). An alternative explanation for higher rates of drug resistance amongst lineage 2 strains may be the founder effect, where lineage 2 strains that were already drug resistant clonally expanded rapidly in an area with high rates of transmission (Grandjean et al., 2015).

Finally, country-specific factors have been shown to influence the development of resistance even within a given bacterial strain. One example from a reconstructed phylogeny of the Central Asian Clade, a subgroup of lineage 2.2 (Beijing strain), showed it was in circulation in former Soviet republics in the 1960s and 1970s, before its introduction into Afghanistan in the 1980s (Eldholm et al., 2016). Many resistance mutations arose independently amongst strains that were circulating in the former Soviet republics, while very few apart from the original lineage-defining *rpoB* mutation were present in the Afghan strains, with the vast majority of these mutations arising in the years after the collapse of the Soviet Union. Another analysis of the global spread of lineage 4 found that it was likely to have been dispersed from Europe during colonial expansion, that most drug resistance conferring mutations arose and were subsequently spread within individual countries (Brynildsrud et al., 2018). Taken together, this suggests that a variety of factors that are hard to quantify, such as differing healthcare systems and political instability that are likely to impact on patterns of antimicrobial prescription, supply and usage. Additionally, country-level variation in sequencing and drug susceptibility testing is also likely to play a significant role in the determined level of resistance in each country. For example, in 2020 94% of new TB cases in the WHO Europe Region were tested for rifampicin resistance, compared to 50% in the African Region (World Health Organization, 2022).

## Emergence of resistance between hosts

Since the introduction of the first antituberculosis drug, streptomycin, in the 1950s, resistance to most new drugs has been identified as occurring within 5 to 10 years of their clinical use, with similar mutations occurring independently in different parts of the world (convergent evolution) (Cohen et al., 2015; Manson et al., 2017; Brynildsrud et al., 2018). This phenomenon has occurred even more rapidly with recently introduced drugs.

**Figure 2:**
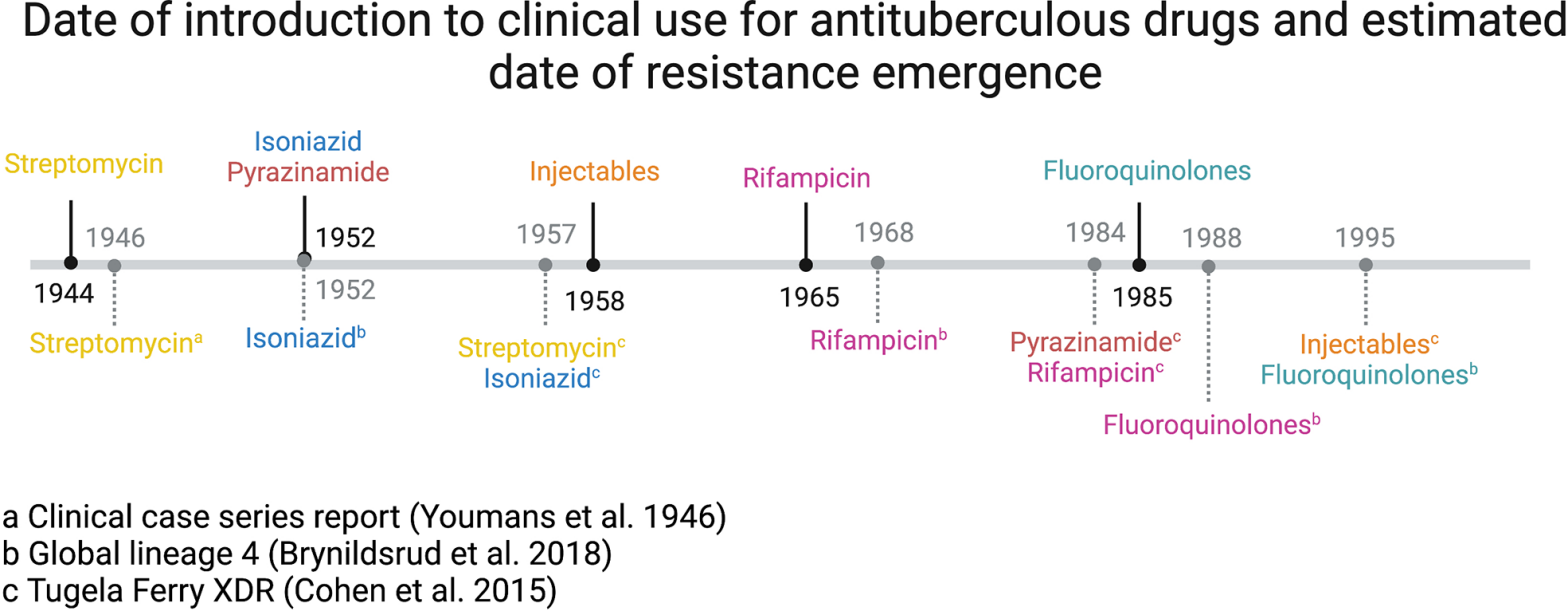
Date of introduction to clinical use for antituberculous drugs (above line, denoted by solid black line) and estimated date of resistance emergence (below line, denoted by grey dashed line) (Ektefaie et al., 2021).

An analysis of the world’s first comprehensively described extensively drug-resistant TB (XDR-TB, by historical definition of injectable and fluoroquinolone resistance) outbreak in Tugela Ferry, KwaZulu-Natal, South Africa, revealed that the drug resistance mutations carried by the strain had been acquired sequentially over 50 years. Genomic dating techniques revealed resistance developing broadly in the order in which drugs were introduced into clinical practice (Cohen et al., 2015), and a similar pattern was demonstrated in a global collection of over 1500 lineage 4 strains (Brynildsrud et al., 2018). This confirms the pattern established after the introduction of streptomycin in the mid-1940s, where clinical resistance was reported within two years (Youmans et al., 1946) (**Figure 2**). Two other studies of multiple global lineages have also shown the same order of resistance development, starting with isoniazid and streptomycin resistance, followed by rifampicin, fluoroquinolones and injectables (Manson et al., 2017; Ektefaie et al., 2021). Interestingly, these studies did not show a correlation between the date of drug introduction and the date of resistance emerging. This is likely to represent the fact that other variables affect the development of resistance, such as the spontaneous rate at which mutations develop, *in vivo* fitness costs associated with resistance, and the clinical combinations in which drugs tended to be used (for example, while injectable drugs have been available since the 1950s they were much less commonly used than rifampicin and isoniazid).

Intriguingly, *Rv0678* mutations likely to confer bedaquiline resistance have been identified long prior to the development of the drug van (van Dorp et al., 2020). It has been hypothesised that this could have been selected for by the use of clofazimine, which was developed for the treatment of TB in the 1950s, although due to the development of more effective TB drugs was mostly used for the treatment of leprosy until it was repurposed for MDR-TB in the 2000s. The earliest emergence was dated to the beginning of the 18^th^ century, although interestingly this clade had an associated inactivating mutation in *mmpL5* which was likely to counteract the resistant phenotype (Sonnenkalb et al., 2021). However, later emergences in the late 19^th^ and early 20^th^ century still pre-date the use of any antituberculosis therapy, and may be due to other environmental stressors, including microbial antagonism in the environment before the transition to obligate pathogenicity. As one role of MmpL5 is efflux – especially of siderophores – this could include adapting to low iron availability or presence of a toxin for example. Overall, this demonstrates that, at least for bedaquiline and clofazimine, and potentially other new drugs, resistant bacteria may already exist in the environment and could be rapidly selected for as therapy is expanded.

## Compensatory mutations

Many TB drugs target essential cellular processes, hence resistance-conferring mutations can come with a fitness cost, manifested as a slower growth rate in culture and reduced transmission within a population compared to wild-type strains. This has been best described for *rpoB* mutations conferring rifampicin resistance (Gagneux et al., 2006; Knight et al., 2015). The most common isoniazid resistance mutation in *katG* (S315T) is thought to have only minimal fitness cost, while pyrazinamide resistance may have a fitness cost (Pečerska et al., 2021). Most fluoroquinolone resistance-conferring mutations do not affect fitness, although impaired growth has been reported for the *gyrA* G88C and G88D mutations, although these occur infrequently in clinical isolates (Emane et al., 2021).

The fitness cost imposed by *rpoB* mutations can be reversed by compensatory mutations, specifically mutations in *rpoA* and *rpoC* which encode two other subunits of the RNA polymerase enzyme (alpha and beta prime). These have been identified in *in vitro* culture experiments and additionally are seen at an increased prevalence in countries with a high burden of MDR-TB (Comas et al., 2011; Merker et al., 2018; Trauner et al., 2021), linked to high rates of transmission of MDR-TB strains in some settings (Gygli et al., 2021).

## Emergence of resistance within hosts

**Figure 3:**
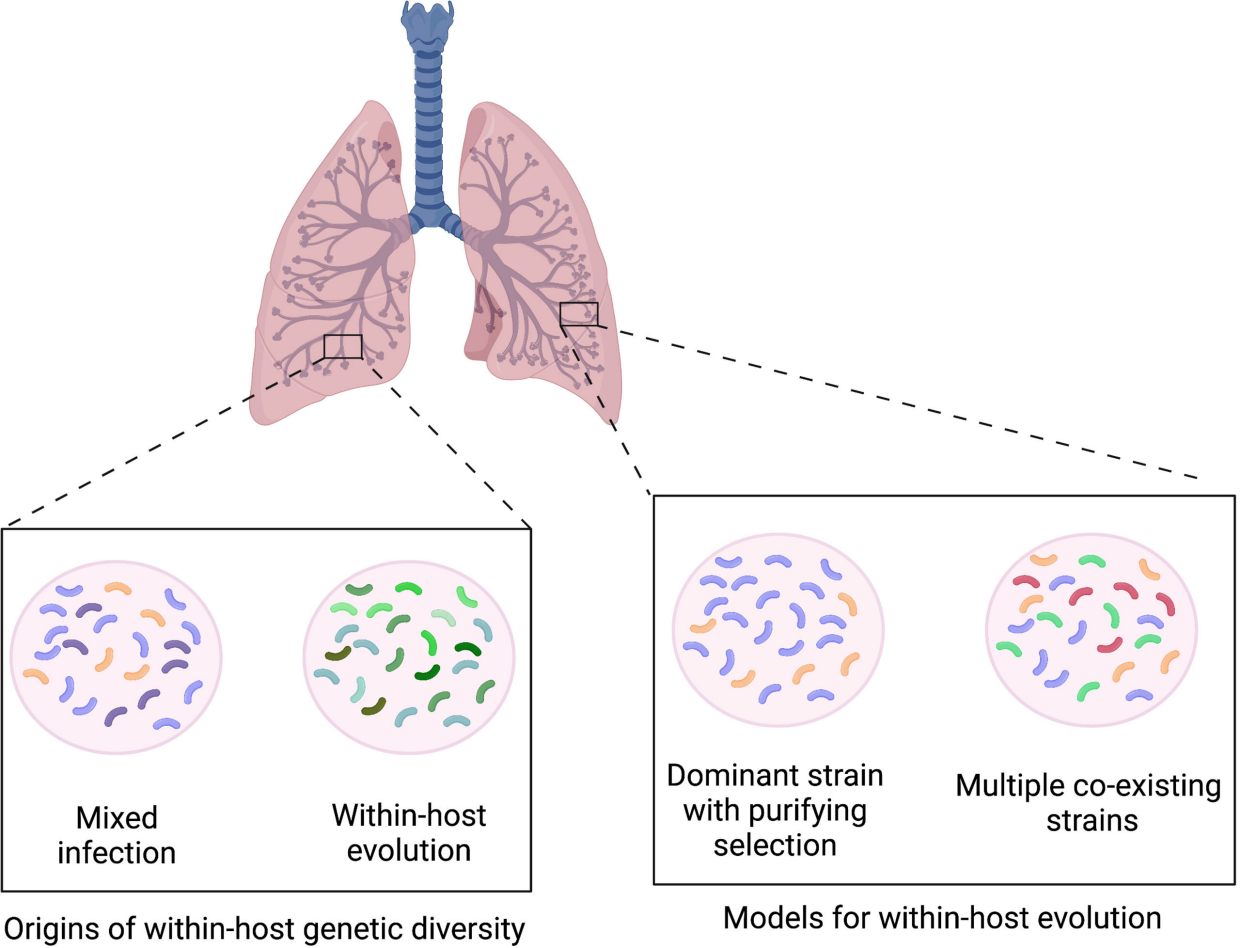
Infection model showing how within-host genetic diversity may occur through mixed infection with genetically different strains or within-host evolution of a clonal infecting strain. Model of within-host evolution showing single dominant strain, with purifying selection leading to loss of variants with reduced fitness and multiple co-existing strains within lung.

It is likely that the ability of *M. tuberculosis* to rapidly acquire drug resistance while maintaining limited genomic variability over time is due the generation of low frequency within-host variation. This is not surprising given that *M. tuberculosis* infections typically last months to years and within-host bacterial populations may peak at over 10^9^ colony forming units. Greater insight into this has been achieved through the adoption of high throughput sequencing, which has more recently enabled the identification significant within-host *M. tuberculosis* genetic diversity. Within-host diversity can in principle arise from mixed infection with multiple genetically distinct strains or within-host microevolution of a single infecting strain, or both (**Figure 3**) (Ford et al., 2012). At one extreme, up to 50 consensus-level SNP differences having been reported to occur over the duration of infection in patients with advanced disease when sampling from multiple body sites (Lieberman et al., 2016). However in the majority of cases of *M. tuberculosis* infection, the genetic diversity is constrained by purifying selection that leads to loss of variants without a clear fitness advantage for this specialised pathogen which is adapted to a pathogenic lifestyle in the human host (**Figure 3**).

Although most *M. tuberculosis* variants are lost over the course of infection, it is still possible for those that confer an advantage, such as drug resistance, to increase in prevalence and become the dominant clone over time. These resistant strains can then be transmitted leading to primary drug resistant infection in a new host. This may contrast with other bacteria where multiple variant strains may co-exist separately, as may be seen in non-specialised pathogens such as *Pseudomonas aeruginosa* (Winstanley et al., 2016) or non-tuberculous mycobacteria (Bryant et al., 2016; Shaw et al., 2019) in patients with cystic fibrosis, where there is an abnormal airway and immune environment (**Figure 3**).

Understanding factors affecting overall within-host *M. tuberculosis* genetic diversity may offer insights into mechanisms controlling bacterial replication and evolution. Most studies to date rely on sequencing mycobacterial DNA extracted from culture to ensure sufficient DNA for sequencing, although this is likely to introduce bias by stochastic loss and selecting for bacterial subpopulations more suited to growth in culture (Metcalfe et al., 2017). However, it has been demonstrated that culture-independent sequencing of *M. tuberculosis* directly from sputum identifies more genetic diversity than sequencing from culture (Nimmo et al., 2019; Shockey et al., 2019). As techniques for direct-from-sample sequencing improve, our understanding of within patient genetic diversity may therefore continue to develop.

From current work that had relied on sequencing from culture, one detailed study of five patients revealed that overall *M. tuberculosis* genomic diversity increased with disease severity and was particularly high in pre-mortem isolates from two patients, presumably due to high bacterial load (O’Neill et al., 2015). The most sequence-diverse genes were those involved in production of cell envelope lipids. No evidence for a decrease in diversity during treatment or any effect of *M. tuberculosis* lineage or drug resistance profile was found, while HIV statuses were not available for analysis. An analysis of 200 patients from eight publicly available studies reporting patients who failed treatment found that genes associated with antibiotic resistance displayed highest diversity, while the within-host diversity across remaining gene classes (*in vitro* essential, non-essential, PE/PPE genes and antigen genes) seemed unaffected (Vargas et al., 2021). South African cohort studies revealed greater genetic diversity in patients with cavitary disease, infection with lineage 2 strains and absence of second-line drug resistance, although no association between time to positivity in culture and diversity (Nimmo et al., 2020). This suggests that diversity may be more influenced by higher intrinsic mutation rates (as seen with lineage 2), variable drug penetration (in cavitary disease) or impaired immune control (in untreated HIV) than bacterial population size. However, there was no association between diversity and clinical outcomes at six months.

Mixed populations of wild-type alleles and resistance-associated variants (RAVs) confer heteroresistance, where populations of resistant and susceptible bacteria co-exist within the same host. This may occur as the result of differential drug penetration to spatially and pathologically distinct lung regions (Dheda et al., 2018) leading in effect to monotherapy and subsequent resistance acquisition or survival of susceptible bacteria. Baseline genetic heteroresistance appears to be particularly common for bedaquiline (up to 60%) (Nimmo et al., 2020) and fluoroquinolones (11-26%) (Operario et al., 2017; Nimmo et al., 2020).

Several case reports have identified heterozygous RAVs that have increased in frequency over the course of treatment (Sun et al., 2012; Eldholm et al., 2014; Trauner et al., 2017) leading to fixed resistance, including variants originally identified at <1% frequency (Vos et al., 2019). A retrospective deep sequencing study identified very low frequency RAVs (<1%) predating acquired phenotypic resistance (Engelthaler et al., 2019). However, due to high levels of turnover of low-frequency variants, it may be difficult to predict which heterozygous RAVs are likely to persist or become fixed and which ones will disappear. In a prospective cohort study of almost 400 patients, most with heterozygous RAVs detectable on WGS with sequential isolates available and sensitivity to detect variants above 5% frequency demonstrated RAV persistence or fixation (17/20, 85%) (Nimmo et al., 2020). However, only one case of a very low frequency RAV (<5%) expanding to cause resistance was identified. Another study showed no effect on treatment outcome amongst patients with RAVs at <1% frequency (Chen et al., 2021), while modelling from multiple cohort studies suggests that variants at ≥19% frequency predicted subsequent fixation (Vargas et al., 2021).

Taken together, the current evidence suggests that the significance of heterozygous RAVs is likely to depend on their frequency, with much greater clinical significance of those at higher frequency (>15-20%) than lower frequency (especially <5%). While heterozygous RAVs are likely to be variably identified by current diagnostics (Ng et al., 2019; Rigouts et al., 2019) with newer sequencing-based techniques offering good sensitivity even for RAVs identified at very low frequency, establishing how to interpret low frequency heterozygous RAVs is going to become an important clinical decision.

## Implications for diagnostics

**Figure 4:**
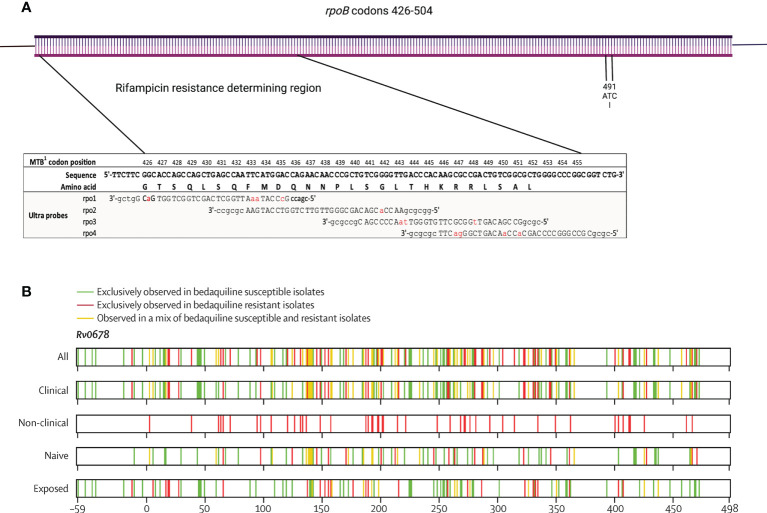
Schematic diagrams of **(A)** *rpoB* gene codons 426-504 showing rifampicin resistance determining region, coverage of Xpert MTB/RIF probes (modified from (Chakravorty et al., 2017) under CC BY 4.0 licence) and location of I491 codon, and **(B)** *Rv0678* gene showing distribution of reported gene substitutions, coloured by association with resistance (reproduced from (Ismail et al., 2021) under CC BY 4.0 licence).

As many countries move towards genomic-based methods such as molecular PCR-based tools (for instance Xpert MTB/RIF), and progressively to targeted sequencing and whole genome sequencing (WGS) for diagnosis of *M. tuberculosis* infection and identification of drug resistance, it is important to be aware of the implications of the evolution of resistance. Currently, understanding of resistance-conferring mutations is inevitably limited, particularly for newer drugs, despite recent large global studies (The CRyPTIC Consortium, 2022). The clearest example of a targeted molecular tool creating its own selective pressure has been demonstrated in Eswatini where the non-canonical rifampicin resistance-conferring mutation, *rpoB* I491F, which falls outside the rifampicin-resistance determining region became dominant (**Figure 4A** **) (** Sanchez-Padilla et al., 2015 **).**

This was first identified following the Eswatini 2009 drug resistance survey, which revealed a surprisingly high rate of MDR-TB (7.7% in previously untreated patients and 33.8% in previously treated patients) (Sanchez-Padilla et al., 2012). Xpert MTB/RIF was implemented in 2012 to enable rapid diagnosis of MDR-TB (Sikhondze et al., 2015), but in 2015 a detailed genetic and phenotypic analysis of strains stored from the 2009 survey showed that 30% of rifampicin resistance was actually conferred by a the *rpoB* I491F mutation, which is not identified by Xpert MTB/RIF and such strains would therefore be reported as rifampicin susceptible (Sanchez-Padilla et al., 2015). Patients infected with such strains would therefore be treated with an ineffective standard drug-susceptible regimen for their rifampicin-resistant infection. By the time of the next drug resistance survey in 2017, 56% of rifampicin resistance was conferred by the I491F mutation (World Health Organization, 2020). The Eswatini National Tuberculosis Control Programme has since proposed presumptively treating all isoniazid-resistant TB as MDR-TB until phenotypic testing has been completed, as most I491F mutations are present in isoniazid resistant strains (Ardizzoni et al., 2021).

Finally, resistance to new drugs such as bedaquiline and delamanid is caused by many individually rare mutations that do not provide statistical power for genotype-phenotype correlation in the way that has been performed for most first-line drugs. For example, in the *Rv0678* gene responsible for most clinical bedaquiline resistance, mutations are spread throughout the gene with no clear resistance-conferring hotspot (**Figure 4B**). Additionally, there is not a clear separation of minimum inhibitory concentrations of bedaquiline between wild type and resistant isolates, which is likely to complicate attempts to categorise individual mutations as susceptible or resistant, with many likely to fall near the critical concentration and be vulnerable to technical variation. A pragmatic approach may be to use molecular or genetic methods to screen resistance-associated genes for variants, which are rare amongst susceptible isolates, followed by phenotypic evaluation of isolates containing mutants.

## Conclusions

*M. tuberculosis* has shown a remarkable ability to develop resistance to all antituberculosis drugs that have been developed, including those brought into clinical use for DR-TB in recent years such as bedaquiline, linezolid and delamanid/pretomanid. It is important to bear in mind these drugs remain highly effective in the vast majority of patients with DR-TB and have undoubtedly been responsible for the major improvements in DR-TB outcomes that have been seen in the last 10 years (World Health Organization, 2022). However, examples such as the spread of XDR-TB across South Africa, first identified as the Tugela Ferry outbreak, and the rapid amplification of the *rpoB* I491F mutation amongst *M. tuberculosis* strains in Eswatini, highlight that this progress cannot be taken for granted (Gandhi et al., 2006; Sanchez-Padilla et al., 2015). Progression towards the World Health Organization’s End TB targets of a 90% reduction in TB transmission between 2015 and 2035 will require strict control of the spread of DR-TB, which require highly effective drugs to be available rapidly to those infected.

To achieve this, important strategic decisions will be required. While there are now a number of exciting new drug candidates progressing through the TB drug development pipeline, their impact will remain uncertain. The effectiveness of currently available drugs depends on limiting the spread of resistance to them. It is therefore questionable whether effective drugs for DR-TB such as bedaquiline and pretomanid should be incorporated into drug-susceptible TB (DS-TB) regimens, which may increase the spread of resistance and reduce their effectiveness for DR-TB, unless their overall benefits to patients with TB and progress towards elimination is outweighed by improvements in DS-TB treatment. This needs to be accounted for when evaluating the results of trials such as SimpliciTB (ClinicalTrials.gov Identifier: NCT03338621), where bedaquiline and pretomanid are used to reduce DS-TB treatment duration from six to four months, which was already been demonstrated to be possible using rifapentine and moxifloxacin (Dorman et al., 2021), or adopting a stratified treatment approach for some patients (Imperial et al., 2018).

In addition, it is essential not to assume susceptibility to drugs and TB programmes should aim to perform susceptibility testing for all drugs that are included in treatment regimens. It is now clearly demonstrated that there is a pre-existing pool of bedaquiline resistance and it can therefore be expected to occur in patients without any clear risk factors for resistance (Beckert et al., 2020; van Dorp et al., 2020; Ismail et al., 2022) Genotypic susceptibility testing is clearly very effective in many cases, but it is important to be aware of the limitations. While the limited genotypic-phenotypic understanding for new drugs such as bedaquiline and delamanid/pretomanid will be one challenge, the spread of *rpoB* I491F shows how significant the selective pressure from diagnostics can be.

The most effective strategy is therefore going to require greater understanding of how resistance develops within patients with a view to preventing its occurrence. This will need to be backed up by preventing spread of resistance through use of up-front susceptibility testing for all drugs – using combined genotypic and phenotypic methods – along with ongoing surveillance for development of resistance and changes to the prevalence of resistance-conferring mutations, and strategic use of available medications to maximise benefit to individual patients as well as the End TB strategy.

## Author contributions

Conceptualisation: CN;Writing – original draft: CN; Writing – review and editing: CN, JaM, VF, JoM, JE, HP, and EJ; Visualisation: CN, VF, JoM, and HP; Supervision: EOJ; All authors contributed to the article and approved the submitted version.

## Funding

This work was supported by the Francis Crick Institute which receives its core funding from Cancer Research UK (CC2169), the UK Medical Research Council (CC2169), and the Wellcome Trust (CC2169).

## Acknowledgments

**Figures 2**, **3** and **4** created with BioRender.com. This research was funded in part by the Wellcome Trust (CC2169). For the purpose of Open Access, the author has applied a CC BY public copyright licence to any Author Accepted Manuscript version arising from this submission. The authors would like to thank Joanna Evans (Systems Chemical Biology of Infection and Resistance Laboratory, Francis Crick Institute) for critically reading the manuscript.

## Conflict of interest

The authors declare that the research was conducted in the absence of any commercial or financial relationships that could be construed as a potential conflict of interest.

## Publisher’s note

All claims expressed in this article are solely those of the authors and do not necessarily represent those of their affiliated organizations, or those of the publisher, the editors and the reviewers. Any product that may be evaluated in this article, or claim that may be made by its manufacturer, is not guaranteed or endorsed by the publisher.
